# Heat/Cold Stress and Methods to Mitigate Its Detrimental Impact on Pork and Poultry Meat: A Review

**DOI:** 10.3390/foods13091333

**Published:** 2024-04-26

**Authors:** Tomasz Lesiów, Youling L. Xiong

**Affiliations:** 1 Department of Agri-Engineering and Quality Analysis, Wroclaw University of Economics and Business, 53-345 Wroclaw, Poland; 2 Department of Animal and Food Sciences, University of Kentucky, Lexington, KY 40546, USA; ylxiong@uky.edu

**Keywords:** heat stress, cold stress, pigs and poultry, transportation

## Abstract

This paper aims to provide an updated review and current understanding of the impact of extreme temperatures—focusing on heat stress (HS)—on the quality of pork and poultry meat, particularly amidst an unprecedented global rise in environmental temperatures. Acute or chronic HS can lead to the development of pale, soft, and exudative (PSE) meat during short transportation or of dark, firm, and dry (DFD) meat associated with long transportation and seasonal changes in pork and poultry meat. While HS is more likely to result in PSE meat, cold stress (CS) is more commonly linked to the development of DFD meat. Methods aimed at mitigating the effects of HS include showering (water sprinkling/misting) during transport, as well as control and adequate ventilation rates in the truck, which not only improve animal welfare but also reduce mortality and the incidence of PSE meat. To mitigate CS, bedding on trailers and closing the tracks’ curtains (insulation) are viable strategies. Ongoing efforts to minimize meat quality deterioration due to HS or CS must prioritize the welfare of the livestock and focus on the scaleup of laboratory testing to commercial applications.

## 1. Introduction

The presence of pale, soft, and exudative (PSE)-like meat in pork and chicken meat is a significant global food quality concern. The processing properties of PSE-like meat, along with methods to improve the processing or mitigate the reduced functionalities, have garnered increasing attention from processors and researchers in the meat and poultry processing industry [1,2,3,4,5,6]. It is also valid for dark, firm, and dry (DFD)-like meat, even though the incidence of DFD meat is less than that of PSE meat [3].

The emphasis on genetic selection has led to the reduced ability of pigs to cope with heat stress (HS). Pigs with improved reproductive traits and lean tissue growth rates exhibit increased metabolic heat production beyond their adaptive capacity [7,8]. According to Mutua et al. [9], compared with pigs produced a few decades ago, current genetic pig lines generate nearly one-fifth more heat, which disposes the animals to HS. On the other hand, the PSE meat incidence in poultry meat results from the combined effects of genetic factors (selection for body weight or muscle development) [10,11] and environmental factors, such as seasonal transitions [12,13], HS [14,15], and the post-slaughter environment [16]. 

The inherent physiological factors responsible for HS and CS have been investigated. The rapid growth rate of poultry, due to genetic selection, modern feeding and management methods, and the disruption of the thermoregulatory system in birds, explains their inability to control heat under fluctuating ambient temperatures and high metabolic rate conditions. The situation is further exacerbated by birds being covered in feathers and having no sweat glands [17,18]. Similarly, susceptibility to HS in pigs is due to the lack of functional sweat glands and the insulating skin layer of subcutaneous fat [15]. High ambient temperatures and humidity stress cattle, pigs, and poultry [19]. 

Various methods are used to mitigate the consequences of HS in terms of pig and poultry production and meat quality. These include altering the animal’s microenvironment to increase the exchange of heat between the animal and its surroundings (cooling options) or feeding management (changes in diet composition and distribution), genetic selection for thermal tolerance [8], and in the case of animal and poultry transport, conducting transportation at night or reducing the transport time [20].

An increase in global environmental temperatures and weather anomalies, which are increasingly occurring in Europe and other continents [5], prompt an examination of how countries with a tropical climate, such as Brazil, handle the phenomenon of meat defects. HS is the most challenging environmental stressor that negatively impacts the quality of pig or poultry meat. Therefore, this paper aims to present the latest state of the art knowledge on the influence of HS during pig and poultry transportation and potential methods to mitigate the detrimental effects on meat quality.

## 2. Heat Stress

As previously mentioned, the stress experienced by birds during capture and transport, coupled with unfavorable conditions during broiler transport—such as the season of the year and the distance between the farm and slaughterhouse—promotes the occurrence of PSE and DFD meat.

HS occurs when the ambient temperature exceeds the animal’s thermoneutral zone (TNZ, defined as the environmental temperature range within which animals use no additional energy to maintain their body temperature) [21]. HS is broadly classified into acute heat stress (AHS), characterized by intense environmental temperatures for a brief period, and chronic heat stress (CHS), involving prolonged exposure to high temperatures.

AHS immediately before slaughter accelerates muscle glycogenolysis, elevates the lactic acid concentration, and rapidly decreases the muscle pH while the carcass is still hot [22]. This results in poultry, pigs, and cattle PSE meat having a lower water-holding capacity (WHC) [23,24,25,26,27]. In contrast, animals subjected to CHS have low muscle glycogen reserves, leading to lower lactic acid production. As a result, DFD meat has a high pH and higher WHC [23,28,29,30]. High seasonal temperatures more significantly affect lipid and protein oxidation and result in greater microbial susceptibility, and the meat has a shortened shelf life [31,32]. Differently, Lu et al. [33] found that CHS (14 days of broilers’ heat exposure) affects meat quality by altering the aerobic metabolism, glycolysis, and intramuscular fat deposition, resulting in low customer acceptability due to the pale meat color with low WHC and increased cook and drip losses [33].

**Figure 1:**
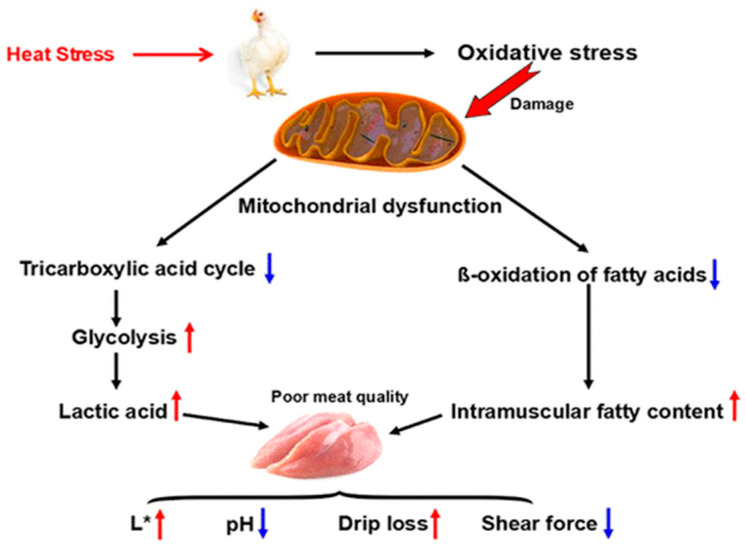
Mechanisms by which HS impairs meat quality by affecting the metabolism of energy substances. Up-pointing red arrow denotes increase; down-pointing blue arrow indicates decrease. Source: Lu et al. [33].

Figure 1 presents the relation between heat stress and the quality of breast muscle broilers. Chronic heat exposure can induce oxidative stress, irreversibly damaging the mitochondria, which are prone to oxidative damage as a result of the high content of phospholipids and proteins in their membranes. CHS also significantly changes the mitochondrial morphology [34] and structure [35]. Mitochondrial dysfunction leads to a decline in the mitochondrial oxidation of energy substrates, such as lipids and carbohydrates, resulting in metabolic changes that affect the meat quality by decreasing the pH_45min_ and shear force (SF) and increasing the L* value and drip loss.

During stress, the depletion of the muscle glycogen reserves induces significant alterations in the final meat pH. Stress during a short pre-slaughter period results in PSE meat, while prolonged stress leads to DFD meat [23,25]. Neither PSE meat with higher exudate and lower texture quality as a result of protein denaturation caused by rapid post-mortem muscle glycolysis nor DFD meat with a dry appearance (water firmly adheres to muscle proteins) are not acceptable [1,3,36,37,38,39].

Zaboli et al. [18] identified three mechanisms explaining AHS or CHS: (1) a rapid pH drop during and after slaughter due to glycogen conversion, leading to increased lactic acid accumulation, especially when the muscle temperature is high, resulting in lower WHC of muscle; (2) acceleration of panting (open-mouth breathing) to dissipate body heat, increasing CO_2_ exhalation and the pH drop in the blood, initiating metabolic acidosis in skeletal muscle; and (3) the reactive oxygen species produced by HS increase oxidative stress in birds, damaging the structure and functions of enzymes that regulate sarcoplasmic calcium levels in muscles, accentuating energy expenditure due to constant muscle contractions.

## 3. Effect of Heat Stress and Transport Time on the Quality of Pork

Čobanovic et al. [40] (Serbia) found that pigs exposed to short transportation durations (approximately 20 min) at a high loading density (0.29 m^2^/100 kg) during hot weather conditions (summer temperatures ranging from 34.5 to 35.2 °C with humidity levels of 87.3–88.8%) produced meat with the lowest initial and ultimate pH values, as well as sensory color scores. Additionally, these pigs had the highest initial temperature and a prevalence of PSE (66.7%) pork [15,41]. The incidence of PSE pork decreased 5-fold during hot weather conditions when the pigs experienced longer transportation durations (approximately 210 min) and a low loading density (0.53 m^2^/100 kg). Conversely, pigs exposed to short transportation durations (approximately 20 min) at a high loading density (0.41 m^2^/100 kg) during cold weather conditions produced higher quality pork, characterized by the highest percentage of red, firm, and non-exudative pork. This pork also showed the lowest drip loss and b* values and the highest sensory color scores. According to Čobanovic et al. [40], the weather conditions and loading density play a more crucial role in carcass damage and pork quality variations than the transportation time.

Gajana et al. [41] (South Africa) found that pigs transported on a lorry for a longer duration (>2 h) in a smaller floor area (0.35 m^2^/100 kg) were more likely to develop PSE meat during the summer (43%) and fall (68%), which are considered hot and humid seasons. Similar observations were made by Küchenmeister et al. [42] and Dalla Costa et al. [43], who noted that temperatures >35 °C had an adverse effect on HS, causing increased PSE meat in pork.

Conversely, high pH values occur when animals experience chronic stress, depleting muscle glycogen rapidly during the pre-slaughter period and after slaughter. This leads to insufficient lactic acid production, resulting in DFD meat [41,44]. Low temperatures during the winter negatively impact meat quality parameters and the incidence of DFD meat. Arduini et al. [45] and Scheeren et al. [46] found that the interaction between longer transportation durations (>2 h) and winter conditions contributed to an increased frequency of occurrence of carcass damage and a higher occurrence of DFD pork (abnormally high ultimate pH, dark color, and increased WHC).

Acute temperature stress is typically induced to mimic the effect of temperature during short-term or daily fluctuations shortly before slaughter (i.e., during transportation or lairage). In contrast, chronic temperature stress simulates the effect of temperature during long-term or seasonal changes that occur during production [47].

## 4. Effects of Heat Stress and Transportation Time on Poultry Meat Quality

Temperature stress, encompassing both hot (HS) and cold conditions (CS), is associated with an increased occurrence of meat quality defects, such as PSE and DFD meat, costing the poultry industry millions of dollars annually. Elevated temperatures lead to increased glycogen breakdown, subsequent acidification, and degradation of muscle protein. In contrast, lower temperatures counteract this effect by depleting glycogen stores before slaughter. Leishman et al. [47] explained that the heightened metabolic demand required to maintain the core body temperature during cold conditions results in the utilization of muscle glycogen as an energy source. The depletion of muscle glycogen before slaughter reduces the pectoralis major (PM) muscle potential for lactate formation in the meat, resulting in a higher muscle pH.

According to Leishman et al. [47], not all birds are susceptible to PSE meat under HS conditions. Although stress susceptibility is well documented in pigs, where susceptible pigs are more likely to develop PSE meat than non-sensitive animals, the genetic cause remains unknown for poultry. In pigs, a point mutation in the ryanodine receptor 1 (RYR1) gene has been determined to be the genetic cause of malignant hyperthermia resulting in PSE meat. In pigs, this gene is sometimes known as the halothane gene (or “HAL” gene) due to the association between porcine stress syndrome and halothane sensitivity. In chickens and turkeys, RYR1 polymorphisms and variants in the RYR1 transcripts have been discovered but not found to be associated with the development of PSE meat. Therefore, the genetic cause of this myopathy in poultry remains unknown. Although the cause has not been pinpointed, it is possible that some birds within a given study treatment are susceptible to PSE meat, while others are not, leading to variation in the effect magnitude between studies. Ensuring effective stress response control during broiler transportation is critical for both animal welfare and meat quality [48,49]. 

**Figure 2:**
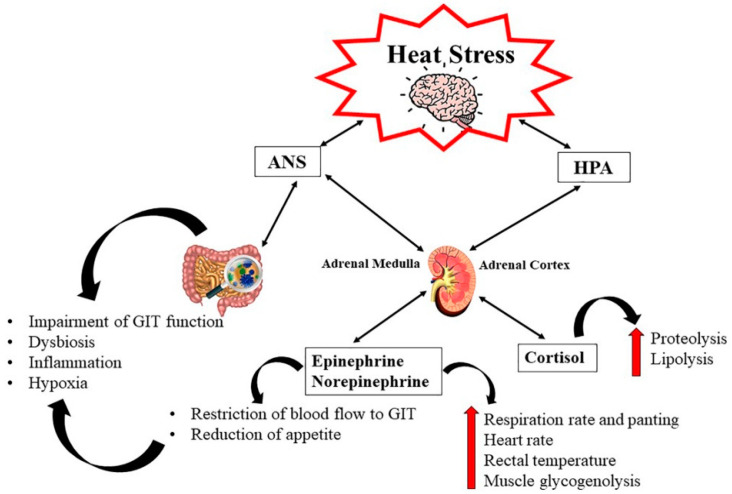
Relationship between HS and the autonomic nervous system (ANS) and the hypothalamic–pituitary–adrenal (HPA) axis. (GIT: gastrointestinal tract). Source: Gonzalez-Rivas et al. [15].

Gonzalez-Rivas et al. [15] explained the stress responses to high ambient temperatures and humidity (Figure 2). These responses primarily involve autonomic reactions through activating the autonomic nervous system (ANS) mediated by catecholamines (adrenaline/epinephrine and noradrenaline/norepinephrine). They include increases in the respiration rate and heart rate, panting, elevated body temperature, redistribution of blood flow from the viscera to the skin for thermoregulation, and promotion of energy utilization from body reserves. This process accelerates muscle glycogenolysis and suppresses energy storage. The authors also reported that AHS and CHS also increase the plasma glucocorticoid concentrations (cortisol) via activation of the hypothalamic–pituitary–adrenal (HPA) axis, which improves heat loss (via vasodilation, increased proteolysis, and changes in lipid metabolism).

Kim and Lee [50] presented a sequence of changes causing the harmful effect of HS in a hen’s intestine, blood vessels and reproductive organs. According to these authors, HS triggers the hypothalamic–pituitary–adrenal (HPA) cortical system, which releases a corticotrophin-releasing factor/hormone (CRH) from the hypothalamus. The hypothalamus sends a message to the pituitary to release an adrenocorticotropic hormone (ACTH), which stimulates the synthesis and leads to an increase in the corticosterone level (CORT).

Genetic selection for rapid muscle growth in poultry has altered the ability of animals to respond and adapt to environmental stressors [15]. Commercial poultry lines could exhibit PSE meat due to muscle hyper-metabolism mediated either by increased Ca^2+^ release from the sarcoplasmic reticulum or by direct action on RYR1, increasing the open state of the channel [15]. HS increases the production of mitochondrial reactive oxygen species in the skeletal muscle of chickens, causing oxidative damage, inducing lipid peroxidation, and leading to oxidative modification of proteins [15].

**Figure 3:**
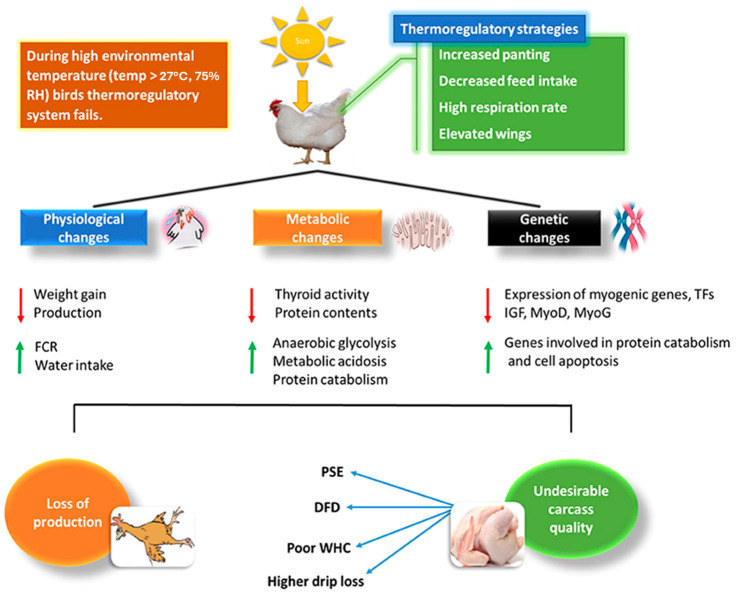
Relationship of HS with physiological and biochemical changes in poultry and how it affects broiler chickens’ meat quality. Up-pointing red arrow denotes increase; down-pointing blue arrow indicates decrease. Source: Nawaz et al. [5].

According to Nawaz et al. [5], high temperatures (exceeding the TNZ, the appropriate temperature for broilers is between 18 and 22 °C [51]) during poultry production phases generate physiological, metabolic, and genetic changes in birds due to HS and harm breeding efficiency and meat quality (Figure 3). Maintaining homeostasis in bird-rearing conditions should be a primary goal, but it is not—that goal is high commercial poultry production. When the temperature rises, the birds maintain homeostasis and need more energy to maintain their body temperature. As previously mentioned, birds do not have sweat glands, and their skin is covered with feathers, so to cope with the heat, birds increase their number of breaths, pant, and raise their wings. However, unable to ensure proper thermoregulation, birds succumb to hyperthermia, which ultimately translates to a loss of production and poorer-quality meat.

## 5. Methods to Mitigate Defects in Pork and Poultry Meat during Transportation in Hot/Ambient Temperatures and Before Slaughter

In pigs, compared to poultry, it is much more difficult for the animal to maintain homeostasis under conditions of high temperature and humidity. In addition to an increased metabolic rate due to genetic selection, there is a thick layer of subcutaneous adipose tissue and relatively small lungs, limiting the effect of getting rid of excess heat through panting [52,53,54].

Interesting observations were made by Machado et al. [55,56] while transporting pigs (weaner pigs) on a 170 km/70 km route in Brazil (ambient temperature 26.8–31.0 °C/28.2–31.2 °C, humidity from 66–72%/62–68%). These authors found that animals located in the lowest part of the vehicle (a vehicle with a trailer) were most vulnerable to heat stress due to the ventilation dynamics, with the front part of the vehicle being the most detrimental to animal welfare. In contrast, pigs transported at the very top of the vehicle were subject to physical stress due to sun exposure and trailer vibration. The authors pointed out that in tropical regions, the practice of “wetting the load” when loading pigs into the lower part of the vehicle due to insufficient ventilation is unfavorable. 

In another study, Machado et al. [57] observed higher production losses due to heat stress during pig transport (semiarid region, afternoon) over a short distance (30 km) than during transport over a longer distance (170 km). In this situation, the animals had time to adjust to the social and physical environment. Therefore, travel planning to determine the coolest hours (e.g., early morning or evening), depending on the vehicle and animal parameters and environmental conditions, is crucial to minimize the HS risk and associated production losses during pig transport.

Only limited information exists regarding the HS experienced by various categories of pigs during transport. Thodberg et al. [58,59] emphasized that in a moderate climatic zone like Denmark, the temperatures in trucks during summer and autumn when transporting sows to slaughter were not maintained within the upper limit of the TNZ appropriate for sows (16–22 °C). Therefore, future studies on the optimization of transport management, ventilation, and logistics should be focused on maintaining temperatures within the comfort zone to mitigate adverse effects on sow welfare during transport.

**Figure 4:**
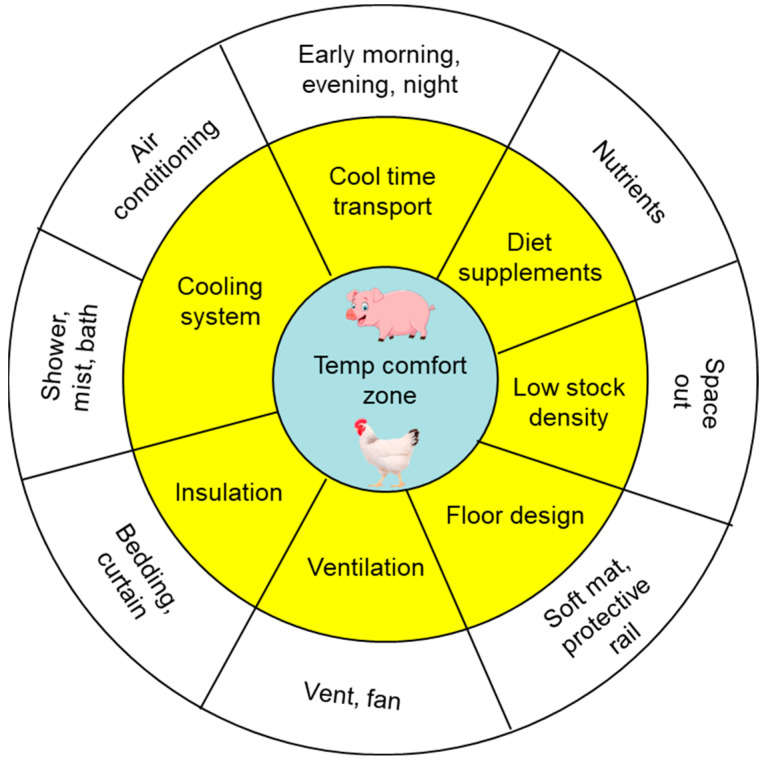
Possible methods to mitigate HS and CS in pigs and poultry during transportation.

Various means and tools have been suggested to mitigate HS and CS during animal transportation. Figure 4 presents some common and potential methods to reduce the incidence of PSE meat in pork and chicken. The exact method to adopt depends on the species (pig or bird), and the cost of a preventative measure is a significant factor. For example, showering, misting, and bathing may be more suitable for poultry due to their small size, while maintaining a relatively low load density and a well-functioning ventilation system are more important for swine species. Often, a combination of different methods, as listed in Figure 4, is applied to reduce extreme temperature stresses and, hence, the PSE and DFD meat incidences.

Rioja-Lan et al. [60] studied the effects of environmental factors on the welfare of transported pigs (100–135 kg) in Canada under conditions of wide temperature fluctuations during the winter (−28.8–1.9 °C) and summer (9.1–40.1° C), which are often outside their thermal comfort zone (10–24 °C). These authors [60] confirmed that the temperatures, air velocities, and humidity in warmer months increase the frequency of HS indicators (e.g., panting, skin discoloration), mortality losses (heart failure), and behavioral and physiological changes in pigs, resulting in poor meat quality. The suggested solutions to reduce the effects of HS or CS are as follows: panting, water sprinkling/misting during transport and lairage, ventilation rates on trucks, night transportation, the amount and type of bedding material used in trailers adjusted according to the season, the use of bedding on trailers in each season, and modified trailer insulation.

Xiong, Gates, and Green-Miller [61] (USA) analyzed the effects of the thermal category, trailer zones, and misting method (before the start of the trip) for mild, warm, and hot weather (16.7–35.3 °C) and cold weather (−10.6–7 °C) transport trips of market pigs. They found that the front top and bottom zones were warmer during cold weather than the rest of the trailer, indicating less ventilation toward the front. Conversely, the conditions were more uniform throughout the trailer for hot temperatures, indicating sufficient ventilation to limit the temperature rise. Misting showed the potential to alleviate high temperatures but resulted in higher humidity index conditions. Moreover, no boarding and bedding combination effect was observed for the spatial distribution of the trailer’s interior temperatures. However, there are still many unanswered questions: How do we design the type of ventilation when transporting animals of different weights (mechanical or passive) and what is the capacity to adapt to local climatic conditions to maintain the optimal temperature for animals? 

Nielsen et al. [20] recently recommended protecting transported pigs from high temperatures and HS. The most important recommendations include placing them at pick-up points with canopies, allowing wind to flow through the facilities, equipping vehicles with fans to aid ventilation, transporting them at night to avoid the hottest hours of the day, and reducing the stocking density during the preparation stage.

Wang et al. [62] offered another solution to reduce the adverse effects of HS during prolonged (>3 h) pre-slaughter transport of poultry in summer. Supplementation with resveratrol [61] at a dose of 400 mg/kg for 21 days or guanidine acetic acid at a dose of 1200 mg/kg for 14 days [49] before slaughtering broilers exposed to HS can help reduce the rate of glycolysis of PM muscles, which mainly contain glycolytic fibers [63], which can improve meat quality. 

Several authors have studied the effect of water-bathing birds just before summer transport to the slaughterhouse and holding broilers before slaughter [13,64,65,66]—Brazil, China. It turned out that water-bathing birds at about 30 °C for 7 min [13] before transport over short distances (over 3 km) harmed the welfare of chicken broilers. On the other hand, this treatment was beneficial over a longer distance, significantly reducing the amount of PSE meat in the summer. Jiang et al. [64,65] also observed that the use of water-misting sprays with ventilation in the projected shed housing and post-transport (1 h, temperature 33–35 °C, relative humidity ~65%) holding birds in quiet, cooler areas (30 min, 30 °C, relative humidity 60%) alleviated and reduced the energy depletion in post-mortem PM muscles caused by HS. Similarly, Zhang et al. [66] showed that water-misting spray and ventilation before and holding treatment after summer transport for broilers improved the fresh meat quality, especially the water-holding capacity (WHC).

**Figure 5:**
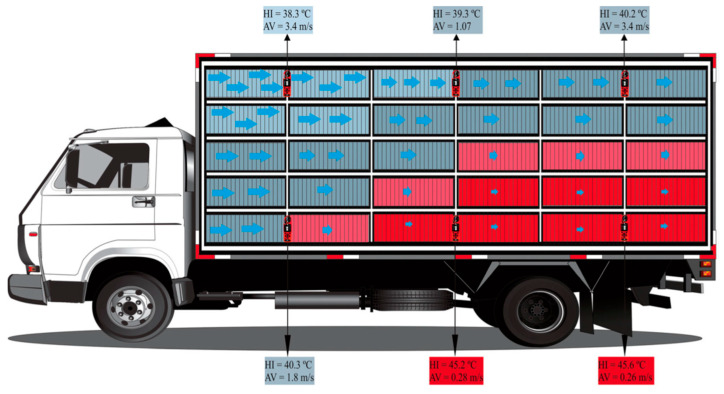
Deduced thermal core formed in a vehicle container under hot–humid conditions (HI heat index, AV air ventilation, airflow pattern 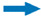). Source: Carvalho et al. [67].

However, using environmentally controlled sheds and modern ventilation systems is a short-term solution because it costs too much for farmers to implement [5]. Therefore, Spurio et al. [14] (Brazil—tropical and subtropical regions, temperature 28.7 ± 2.6 °C, and relative humidity 53.8 ± 9%) found a low-cost solution for improving animal welfare conditions and meat quality. They designed [14] truck containers that allowed for a reduction in the occurrence of PSE meat by 66.3% and 49.6% with and without wetting, respectively. In another study, Carvalho et al. [67] evaluated the effect on turkey welfare in the HS conditions of routine practices in truck transportation. They focused on the heat distribution in trucks (Figure 5). They found a thermal core formed in the middle and rear of the truck container regions; the heat was produced by the birds and the birds in these regions were more prone to a higher incidence of dead-on-arrival (DOA) index and PSE-like meat occurrence. A water-shower treatment applied to the birds at the farm and adequate ventilation throughout the vehicle due to a better truck container design are challenges to improving animal welfare and reducing the mortality and PSE meat incidence.

Birds are sensitive to cold temperatures. Windchill combined with low temperatures can cause thermal discomfort in birds (values below 23 and 29 °C [68] or 18 and 30 °C [69]), and such hypothermal conditions deplete muscle glycogen to maintain body warmth. Carvalho et al. [70] found that the turkey breast muscles of birds transported (38 ± 10 km) in an open vehicle container in the Brazilian winter season conditions (temperature: 3–7 °C, relative humidity: 45–55%), located in the interior compartments of the middle and rear truck regions and subjected to water-bathing treatment, had the highest DFD-like and the lowest PSE-like meat incidence. Therefore, CS related to weather conditions can be reduced during transportation on cold days and nights by closing the tracks’ curtains [48] (Belgium).

## 6. Conclusions

It is expected that efforts will soon be undertaken to improve animal welfare during rearing, transport, and pre-slaughter operations. Weather conditions and the loading density during transportation are more critical factors influencing carcass damage and pork quality variations than the transportation time alone. Nevertheless, transport during the warmest periods (e.g., afternoon) and short-distance journeys (30 km) have been found to result in higher production losses. On the other hand, longer trips (170 km) have allowed pigs to adapt to the social and physical environments they encounter.

Methods aimed at mitigating the effects of HS and CS on poor pork quality include showering (water sprinkling/misting) during transport and lairage, adjusting the ventilation rates in trucks, implementing night transportation, and varying the amount and type of bedding material used in trailers according to the season. Additionally, considering the use of bedding in trailers in each season or modifying trailer insulation can be beneficial.

For chicken broilers, the strategies to reduce meat quality loss due to HS involve dietary resveratrol supplementation on the farm, bathing birds with water just before the journey from the farm to the slaughterhouse for a relatively long distance, utilizing water-misting spray and ventilation before and holding treatment after transport, and improving truck container design to enhance the microenvironment and ensure adequate ventilation throughout the vehicle.

While transporting large slaughter animals and poultry to meat plants, the current methods to reduce HS on the animals and birds could be further refined. To date, research on improving the conditions for the transport of animals and birds in warmer weather through measures such as the air supply and cage rotations has yielded benefits. However, these improvements have not yet been implemented on a practical, mass scale. Due to the environmental impact of raising pigs, cattle, and poultry, a shift in eating habits and relying on genetic attempts to produce a meat-like product from plant raw materials could potentially reduce meat production and make the raw material more expensive. This alternative might lead to a resurgence of traditional animal-raising methods and a departure from mass-scale production.

## Data Availability

No new data were created or analyzed in this study. Data sharing is not applicable to this article.

## References

[1] Barbut S., Sosnicki A.A., Lonergan S.M., Knapp T., Ciobanu D.C., Gatcliffe L.J., Huff-Lonergan E., Wilson E.W. (2008). Progress in reducing the pale, soft and exudative (PSE) problem in pork and poultry meat. Meat Sci..

[2] Dong M., Chen H., Zhang Y., Xu Y., Han M., Xu X., Zhou G. (2020). Processing properties and improvement of Pale, Soft, and Exudative-Like chicken meat: A review. Food Bioprocess Technol..

[3] Lesiów T., Kijowski J. (2003). Impact of PSE and DFD meat on poultry processing—A review. Pol. J. Food Nutr. Sci..

[4] Lesiow T., Rentfrow G.K., Xiong Y.L. (2017). Polyphosphate and myofibrillar protein extract promote transglutaminase-mediated enhancements of rheological and textural properties of PSE pork meat batters. Meat Sci..

[5] Nawaz A.H., Amoah K., Leng Q.Y., Zheng J.H., Zhang W.L., Zhang L. (2021). Poultry response to heat stress: Its physiological, metabolic, and genetic implications on meat production and quality including strategies to improve broiler production in a warming world. Front. Vet. Sci..

[6] Xing T., Xu X., Jiang N., Den S.L. (2016). Effect of transportation and pre-slaughter water shower spray with resting on AMP-activated protein kinase, glycolysis and meat quality of broilers during summer. Anim. Sci. J..

[7] Gourdine J.-L., Rauw W.M., Gilbert H., Poullet N. (2021). The genetics of thermoregulation in pigs: A review. Front. Vet. Sci..

[8] Mayorga E.J., Renaudeau D., Ramirez B.C., Ross J.W., Baumgard L.H. (2019). Heat stress adaptations in pigs. Anim. Front..

[9] Mutua J., Marshall K., Paul B., Notenbaert A.A. (2020). A methodology for mapping current and future heat stress risk in pigs. Animal.

[10] Chen H., Wang H., Qi J., Wang M., Xu X., Zhou G. (2018). Chicken breast quality—Normal, pale, soft and exudative (PSE) and woody—Influences the functional properties of meat batters. Int. J. Food Sci. Technol..

[11] Petracci M., Cavani C. (2012). Muscle growth and poultry meat quality issues. Nutrients.

[12] Carvalho R.H., Soares A.L., Honorato D.C.B., Guarnieri P.D., Pedrão M.R., Paião F.G., Oba A., Ida E.I., Shimokomaki M. (2014). The incidence of pale, soft, and exudative (PSE) turkey meat at a Brazilian commercial plant and the functional properties in its meat product. LWT—Food Sci. Technol..

[13] Langer R.O.d.S., Simões G.S., Soares A.L., Oba A., Rossa A., Shimokomaki M., Ida E.I. (2010). Broiler transportation conditions in a Brazilian commercial line and the occurrence of breast PSE (pale, soft, exudative) meat and DFD-like (dark, firm, dry) meat. Braz. Arch. Biol. Technol..

[14] Spurio R.S., Soares A.L., Carvalho R.H., Silveira Junior V., Grespan M., Oba A., Shimokomaki M. (2016). Improving transport container design to reduce broiler chicken PSE (pale, soft, exudative) meat in Brazil. Anim. Sci. J..

[15] Gonzalez-Rivas P.A., Chauhan S.S., Ha M., Fegan N., Dunshea F.R., Warner R.D. (2020). Effects of heat stress on animal physiology, metabolism, and meat quality: A review. Meat Sci..

[16] Carvalho R.H., Soares A.L., Grespan M., Spurio R.S., Coró F.A.G., Oba A., Shimokomaki M. (2015). The effects of the dark house system on growth, performance and meat quality of broiler chicken: Dark house and broiler meat. Anim. Sci. J..

[17] Loyau T., Berri C., Bedrani L., Metayer-Coustard S., Praud C., Duclos M.J., Tesseraud S., Ridea N., Everaert N., Yahav S. (2013). Thermal manipulation of the embryo modifies the physiology and body composition of broiler chickens reared in floor pens without affecting breast meat processing quality. J. Anim. Sci..

[18] Zaboli G., Huang X., Feng X., Ahn D.U. (2019). How can heat stress affect chicken meat quality?—A review. Poult. Sci..

[19] Buckham-Sporer K., Earley B., Marti S. (2023). Current knowledge on the transportation by road of cattle, including unweaned calves. Animals.

[20] Nielsen S.S., Alvarez J., Bicout D.J., Calistri P., Canali E., Drewe J.A. (2022). Welfare of pigs during transport. EFSA J..

[21] Zhang M., Dunshea F.R., Warner R.D., DiGiacomo K., Osei-Amponsah R., Chauhan S.S. (2020). Impacts of heat stress on meat quality and strategies for amelioration: A review. Int. J. Biometeorol..

[22] Owens C.M., Alvarado C.Z., Sams A.R. (2009). Research developments in pale, soft, and exudative turkey meat in North America. Poult. Sci..

[23] Adzitey F., Huda N. (2011). Pale soft exudative (PSE) and dark firm dry (DFD) meats: Causes and measures to reduce these incidences—A mini review. Int. Food Res. J..

[24] Freitas A.S., Carvalho L.M., Soares A.L., Oliveira M.E., Madruga M.S., Neto A.C., Carvalho R.H., Ida E.I., Shimokomak M. (2017). Pale, soft and exudative (PSE) and dark, firm and dry (DFD) meat determination in broiler chicken raised under tropical climate management conditions. Int. J. Poult. Sci..

[25] Santos V.M., Dallago B.S.L., Racanicci A.M.C., Santana Â.P., Bernal F.E.M. (2017). Effects of season and distance during transport on broiler chicken meat. Poult. Sci..

[26] Kim Y.H.B., Warner R.D., Rosenvold K. (2014). Influence of high pre-rigor temperature and fast pH fall on muscle proteins and meat quality: A review. Anim. Prod. Sci..

[27] Warner R.D., Dunshea F.R., Gutzke D., Lau J., Kearney G. (2014). Factors influencing the incidence of high rigor temperature in beef carcasses in Australia. Anim. Prod. Sci..

[28] Kadim I.T., Mahgoub O., Al-Marzooqi W., Al-Ajmi D.S., Al-Maqbali R.S., Al-Lawati S.M. (2008). The influence of seasonal temperatures on meat quality characteristics of hot-boned, m. psoas major and minor, from goats and sheep. Meat Sci..

[29] Mitlöhner F.M., Galyean M.L., McGlone J.J. (2002). Shade effects on performance, carcass traits, physiology, and behavior of heat-stressed feedlot heifers. J. Anim. Sci..

[30] D’Souza D.N., Leury B.J., Dunshea F.R., Warner R.D. (1998). Effect of on-farm and pre-slaughter handling of pigs on meat quality. Aust. J. Agric. Res..

[31] Mujahid A., Pumford N.R., Bottje W., Nakagawa K., Miyazawa T., Akiba Y., Mitochondrial M. (2007). Oxidative damage in chicken skeletal muscle induced by acute heat stress. J. Poult. Sci..

[32] Wang R.R., Pan X.J., Peng Z.Q. (2009). Effects of heat exposure on muscle oxidation and protein functionalities of pectoralis majors in broilers. Poult. Sci..

[33] Lu Z., He X., Ma B., Zhang L., Li J., Jiang Y., Zhou G., Gao F. (2017). Chronic heat stress impairs the quality of breast-muscle meat in broilers by affecting redox status and energy-substance metabolism. J. Agric. Food Chem..

[34] Lu Z., He X., Ma B., Zhang L., Li J., Jiang Y., Zhou G., Gao F. (2019). Dietary taurine supplementation improves breast meat quality in chronic heat-stressed broilers via activating the Nrf2 pathway and protecting mitochondria from oxidative attack. J. Sci. Food Agric..

[35] Liu B., Jiang J., Yu D., Lin G., Xiong Y.L. (2021). Cellular antioxidant mechanism of selenium-enriched yeast diet in the protection of meat quality of heat-stressed hens. Food Biosci..

[36] Swatland H.J. (1995). On-Line Evaluation of Meat.

[37] Olivo R., Soares A.L., Ida E.I., Shimokomaki M. (2007). Dietary vitamin E inhibits poultry PSE and improves meat functional properties. J. Food Biochem..

[38] Petracci M., Mudalal S., Soglia F., Cavani C. (2015). Meat quality in fast-growing broiler chickens. World’s Poult. Sci. J..

[39] Pietrzak M., Greaser M.L., Sosnicki A.A. (1997). Effect of rapid rigor mortis processes on protein functionality in pectoralis major muscle of domestic turkeys. J. Anim. Sci..

[40] Čobanovic N., Novaković S., Tomašević I., Karabasil N. (2021). Weather conditions, transportation time and loading density and meat quality. Arch. Anim. Breed..

[41] Gajana C.S., Nkukwana T.T., Marume U., Muchenje V. (2013). Effects of transportation time, distance, stocking density, temperature and lairage time on incidences of pale soft exudative (PSE) and the physico-chemical characteristics of pork. Meat Sci..

[42] Küchenmeister U., Kuhn G., Ender K. (2000). Seasonal effects on Ca^2+^ transport of sarcoplasmic reticulum and on meat quality of pigs with different malignant hyperthermia status. Meat Sci..

[43] Dalla Costa O.A., Faucitano L., Coldebella A., Ludke J.V., Peloso J.V., Dalla Roza D., Paranhos da Costa M.J.R. (2007). Effects of the season of the year, truck type and location on truck on skin bruises and meat quality in pigs. Livest. Sci..

[44] Čobanović N., Karabasil N., Stajković S., Ilić N., Suvajdžić B., Petrović M., Teodorović V. (2016). The influence of pre-mortem conditions on pale, soft and exudative (PSE) and dark, firm and dry (DFD) pork meat. Acta Vet.-Beograd..

[45] Arduini A., Redaelli V., Luzi F., Dall’Olio S., Pace V., Costa L.N. (2014). Effect of transport distance and season on some defects of fresh hams destined for DPO production. Animals.

[46] Scheeren M.B., Gonyou H.W., Brown J., Weschenfelder A.V., Faucitano L. (2014). Effects of transport time and location within truck on skin bruises and meat quality of market weight pigs in two seasons. Can. J. Anim. Sci..

[47] Leishman E.M., Ellis J., Staaveren N., Barbut S., Vanderhout R.J., Osborne V.R., Wood B.J., Harlander-Matauschek A., Baes C.F. (2021). Meta-analysis to predict the effects of temperature stress on meat quality of poultry. Poult. Sci..

[48] Jacobs L., Delezie E., Duchateau L., Goethals K., Tuyttens F.A.M. (2017). Impact of the separate pre-slaughter stages on broiler chicken welfare. Poult. Sci..

[49] Zhang L., Li J.L., Wang X.F., Zhu X.D., Gao F., Zhou G.H. (2019). Attenuating effects of guanidinoacetic acid on preslaughter transport-induced muscle energy expenditure and rapid glycolysis of broilers. Poult. Sci..

[50] Kim D.H., Lee K.W. (2023). An update on heat stress in laying hens. World’s Poult. Sci. J..

[51] Gouda A., Tolba S., Mahrose K., Felemban S.G., Khafaga A.F., Khalifa N.E., Jaremko M., Moustafa M., Alshaharni M.O., Algopish U. (2024). Heat shock proteins as a key defense mechanism in poultry production under heat stress conditions. Poult. Sci..

[52] Ross J.W., Hale B.J., Seibert J.T., Romoser M.R., Adur M.K., Keating A.F., Baumgard L.H. (2017). Physiological mechanisms through which heat stress compromises reproduction in pigs. Mol. Rep. Dev..

[53] D’Allaire S., Drolet R., Brodeur D. (1996). Sow mortality associated with high ambient temperatures. Can. Vet. J..

[54] Ross J.W., Hale B.J., Gabler N.K., Rhoads R.P., Keating A.F., Baumgard L.H. (2015). Physiological consequences of heat stress in pigs. Anim. Prod. Sci..

[55] Machado N.A.F., Barbosa-Filho J.A.D., Geraldo L.B., Ramalho G.L.B., Pandorfi H., Silva I.J.O. (2021). Trailer heat zones and their relation to heat stress in pig transport. Eng. Agríc..

[56] Machado N.A.F., Martin J.E., Barbosa-Filho J.A.D., Dias C.T.S., Pinheiro D.G., de Oliveira K.P.L., Souza-Junior J.B.F. (2021). Identification of trailer heat zones and associated heat stress in weaner pigs transported by road in tropical climates. J. Therm. Biol..

[57] Machado N.A.F., Barbosa-Filho J.A.D., Martin J.E., da Silva I.J.O., Pandorfi H., Gadelha C.R.F., Souza-Junior J.B.F., Parente M.M.O., Marques J.I. (2022). Effect of distance and daily periods on heat-stressed pigs and pre-slaughter losses in a semiarid region. Int. J. Biometeorol..

[58] Thodberg K., Fogsgaard K.K., Herskin M.S. (2019). Transportation of cull sows—Deterioration of clinical condition from departure and until arrival at the slaughter plant. Front. Vet. Sci..

[59] Thodberg K., Foldager L., Fogsgaard K.K., Gaillard C., Herskin M.S. (2022). Temperature conditions during commercial transportation of cull sows to slaughter. Comput. Electron. Agric..

[60] Rioja-Lang F.C., Brown J.A., Brockhoff E.J., Faucitano L. (2019). A review of swine transportation research on priority welfare issues: A Canadian perspective. Front. Vet. Sci..

[61] Xiong Y., Gates R.G., Green-Miller A.R. (2018). Factors affecting trailer thermal environment experienced by market pigs transported in the US. Animals.

[62] Wang X., Li J., Cong J., Chen X., Zhu X., Zhang L., Gao F., Zhou G. (2017). Preslaughter transport effect on broiler meat quality and post-mortem glycolysis metabolism of muscles with different fiber types. J. Agric. Food Chem..

[63] Zhang C., Wang L., Zhao X.H., Chen X.Y., Yang L., Geng Z.Y. (2017). Dietary resveratrol supplementation prevents transport-stress-impaired meat quality of broilers through maintaining muscle energy metabolism and antioxidant status. Poult. Sci..

[64] Jiang N., Xing T., Han M., Deng S., Xu X. (2016). Effects of water-misting sprays with forced ventilation on post mortem glycolysis, AMP-activated protein kinase and meat quality of broilers after transport during summer. Anim. Sci. J..

[65] Jiang N., Wang P., Xing T., Han M., Xu X. (2016). An evaluation of the effect of water-misting sprays with forced ventilation on the occurrence of pale, soft, and exudative meat in transported broilers during summer: Impact of the thermal microclimate. J. Anim. Sci..

[66] Zhang L., Huang H., Wang P., Xing T., Xu X. (2020). Water-spraying forced ventilation during holding improves the water holding capacity, impedance, and microstructure of breast meat from summer-transported broiler chickens. Poult. Sci..

[67] Carvalho R.H., Honorato D.C.B., Guarnieri P.D., Soares A.L., Pedrão M.R., Oba A., Paião F.G., Ida E.I., Shimokomaki M. (2018). Assessment of turkey vehicle container microclimate on transit during summer season conditions. Int. J. Biometeorol..

[68] Meltzer A. (2007). Thermoneutral zone and resting metabolic rate of broilers. Br. Poult. Sci..

[69] Pereira D.F., Nääs I.A. (2008). Estimating the thermoneutral zone for broiler breeders using behavioral analysis. Comput. Electron. Agric..

[70] Carvalho R.H., Honorato D.C.B., Guarnieri P.D., Soares A.L., Pedrão M.R., Oba A., Paião F.G., Ida E.I., Shimokomaki M. (2017). In-transit development of color abnormalities in turkey breast meat during winter season. J. Anim. Sci. Technol..

